# Obesity as a Potential Risk Factor for Blepharoptosis: The Korea National Health and Nutrition Examination Survey 2008-2010

**DOI:** 10.1371/journal.pone.0131427

**Published:** 2015-07-10

**Authors:** Ji-Sun Paik, Su-Kyung Jung, Kyung-Do Han, Sang-Duck Kim, Yong-Moon Park, Suk-Woo Yang

**Affiliations:** 1 Department of Ophthalmology and Visual Science, Seoul St. Mary’s Hospital, College of Medicine, The Catholic University of Korea, Seoul, Korea; 2 Department of Biostatistics, College of Medicine, The Catholic University of Korea, Seoul, Korea; 3 Department of Ophthalmology, Wonkwang University, College of Medicine, Iksan, Korea; 4 Department of Epidemiology and Biostatistics, Arnold School of Public Health, University of South Carolina, 800 Sumter Street, Columbia, South Carolina, 29208, United States of America; Save Sight Institute, AUSTRALIA

## Abstract

**Purpose:**

To examine obesity parameters as potential risk factors associated with blepharoptosis in a representative Korean population.

**Methods:**

We analyzed the Korea National Health and Nutrition Examination Survey (KNHANES), conducted between 2008 and 2010. 10,285 Korean adults (4,441 men and 5,844 women) aged 40 years or older was enrolled. We compared body mass index (BMI), waist circumference (WC) and percentage body fat (BF), according to the severity of blepharoptosis. Multiple logistic regression analysis was conducted to examine the associations of each obesity parameter with blepharoptosis.

**Results:**

The overall prevalence of age-related blepharoptosis was 14.8 % in South Korea. There were significant and graded associations between increasing blepharoptosis severity and the mean value of obesity parameters (P for trend < 0.05). As marginal reflex distance 1 (MRD1) decreased, the prevalence of general obesity and overweight status increased (P for trend=0.121 in men and < 0.001 in women); the prevalence of abdominal obesity increased (P for trend < 0.001 for both genders); the prevalence of highest quartile of percentage BF increased (P for trend ≤0.001 for both genders). Blepharoptosis was significantly associated with general obesity in women (adjusted odds ratio (aOR), 2.14; 95% confidence intervals (CI), 1.32-3.47); and with the highest quartile of percentage BF in men (aOR, 2.01; 95% CI, 1.34-2.97) and in women (aOR, 1.52; 95% CI, 1.06-2.3317, after adjusting for age, smoking exercise, drinking alcohol, total energy intake, fat intake, total cholesterol, and high density lipoprotein cholesterol, diabetes, hypertension, and family history of eye disease.

**Conclusions:**

The etiology of age-related blepharoptosis may be multifactorial and is unclear. Our results suggest that obesity parameters such as BMI, WC and percentage BF might be potential risk factors for age-related blepharoptosis in a representative Korean population.

## Introduction

Age-related blepharoptosis is generally thought to develop from an aponeurotic defect of the levator palpebrae superioris muscle [1]. This might be caused by age-related changes in the eyelid and orbit including degenerative fatty changes in the aponeurosis and fat pad of the upper eyelid. This degeneration often occurs in even the levator muscle [2].

Histopathological findings of involutional blepharoptosis have shown significant fatty infiltration of the levator muscle [2]. It has been thought that the carotenoid contents of the preaponeurotic fat pad may affect the occurrence of involutional blepharoptosis [3]. Thus, several investigators have examined the importance of fatty degeneration and fatty component infiltration of the upper eyelid elevators and the potential role of orbital fat alterations in the development of involutional blepharoptosis [2,4,5].

Several case reports have revealed that general obesity is accompanied by blepharoptosis in congenital or genetic syndromes such as MOMES syndrome [6], floppy eyelid syndrome [7], and Prader-Willi syndrome [8]. However, previously reported studies have not addressed any fat-related risk factors of blepharoptosis, and a recent report showed that dyslipidemia is associated with the presence of age-related involutional blepharoptosis [9]. Generally, obesity is thought to be concomitant with dyslipidemia, as identified in South Asians in a recent report [10]. The author hypothesized that a high body mass index (BMI) and percentage body fat (BF), which are closely associated with metabolic syndrome (such as dyslipidemia), might be involved in the pathogenesis of blepharoptosis, especially age-related blepharoptosis. To date, there has been no previous large population-based study of the relationship between obesity and blepharoptosis. The current study was conducted to examine the association of BMI, waist circumference (WC) and percentage BF, with age-related blepharoptosis in a representative Korean population.

## Materials and Methods

### Subjects

This cross-sectional study was based on data acquired in the KNHANES 2008–2010, representing data from the second year of the KNHANES IV (2007–2009) survey to the first year of the KNHANES V (2010–2012) survey. The KNHANES surveys are conducted annually using a rolling sampling design that involves a complex, stratified, multistage, probability-cluster survey of a representative sample of the noninstitutionalized civilian population in South Korea. The survey was performed by the Korean Ministry of Health and Welfare and had three components: a health interview survey, a health examination survey, and a nutrition survey. Additional details regarding the study design and methods are provided elsewhere [11,12]. There were 11,723 subjects aged 40 years or older among those who participated in the survey between January 2008 and December 2010. We excluded participants with liver cirrhosis, chronic liver disease (n = 69), renal disease (n = 602), or malignancy (n = 215). We also excluded those with missing data for variables included in the analysis (n = 552). After excluding the ineligible subjects, the total number of participants for this analysis was 10,285 (4,441 men and 5,844 women). This survey was reviewed and approved by the Institutional Review Board of the Korea Centers for Disease Control and Prevention, and all participants provided written informed consent.

### Definitions of blepharoptosis

Blepharoptosis was defined as the presentation of a marginal reflex distance 1 (MRD1) of < 2 mm [9,13,14]. The MRD1 is the measurement from the central upper eyelid to the pupillary light reflex [13,14]. Measurement of the MRD1 was performed as follows: positioned at the physician’s eye level, participants were asked to look straight ahead and relax while focusing on a distant target. The physician shined a penlight into the participant’s eye, and the distance from the corneal light reflex to the upper eyelid margin was measured in millimeters [13]. All participants’ lid positions were examined by specially trained team examiners who had been working at ophthalmologic resident physicians over 3 years as one of routine ophthalmologic measures. The Quality of the ophthalmic survey was verified by the Epidemiologic Survey Committee of the Korean Ophthalmologic Society. The ophthalmologic residents participating in this survey are required to complete a training course and to conduct supervised practice before working in the survey field. The use of standardized protocol and the periodic training of examiners by acting staff members of the Committee helped to control the quality and validate results. The differential diagnosis of blepharoptosis was made with particular attention to pseudoptosis associated with eyebrow ptosis and dermatochalasis.

### Anthropometric measurements and body composition analyses

Anthropometric measurements of all participants were performed by specially trained examiners. Body weight and height were measured with the subject barefoot and wearing light clothing and were used to calculate the BMI. The WC was measured to the nearest 0.1 cm in the horizontal plane at the level of the midpoint between the iliac crest and the costal margin at the end of a normal expiration. The percentage of body fat (BF, fat mass/total mass ×100) was measured using dual-energy X-ray absorptiometry systems (DXA; QDR 4500A, Hologic Inc., Waltham, MA, USA) located in the mobile examination centers.

### Statistical analyses

Statistical analyses were conducted using SAS (version 9.3 for Windows; SAS Institute, Cary, NC, USA) survey procedures, to reflect the complex sampling design with the sampling weights of KNHANES, and to provide nationally representative prevalence estimates. A two-sided P-value of <0.05 was considered statistically significant. Baseline characteristics of participants according to gender and presence or absence of blepharoptosis are shown as mean ± standard error (SE) for continuous variables or as proportions (% SE) for categorical variables. Student’s t-test or the chi-square test was used to examine the differences in participant characteristics according to the presence or absence of age-related blepharoptosis. The mean values of obesity parameters including BMI, WC and percentage BF were compared using the general linear model according to the severity of blepharoptosis after adjusting for age, smoking status, physical activity, diabetes mellitus and high blood pressure. In addition, the P for linear trend about the relationship between blepharoptosis and obesity parameters (BMI, WC, and percentage body fat) was obtained Multiple logistic regression analysis was conducted to determine the odds ratios (ORs) and 95% confidence intervals (CI) for association of each specific obesity parameter (BMI, WC, and percentage body fat) with blepharoptosis in which a MRD1 of < 2 mm was used as a dependent variable. The relationships were explored after adjusting for the following confounders: model 1 was adjusted for age: model 2 adjusted for family history of eye disease and life-style related risk factors including smoking, exercise, drinking alcohol, total energy intake, and fat intake along with age: model 3 added an adjustment for clinical parameters including total cholesterol, and high density lipoprotein cholesterol, diabetes, and hypertension. We classified BMI into four categories (obesity; BMI ≥ 25 kg/m^2^, overweight; BMI 23~25 kg/m^2^, normal weight; 18.5~23 kg/m^2^, and underweight; BMI < 18.5 kg/m^2^)[15] and percentage body fat into quartiles (Q1 represented the 25% of subjects with the lowest body fat percentages and Q4 represented the 25% of subjects with the highest body fat percentages). We defined abdominal obesity as a WC ≥ 90 cm in males and ≥ 80 cm in females, according to the WC cut-off values from the recommendation of International Diabetes Federation [16, 17].

## Results

### Baseline characteristics (Table 1)

**Table 1:** Characteristics of study participants according to the presence or absence of age-related blepharoptosis. Data are presented as mean ± SE (for continuous variables) or, % (SE) (for categorical variables). BMI, body mass index; HOMA-IR, homeostasis model assessment-insulin resistance; BP, blood pressure; HDL-C, high density lipoprotein cholesterol; TG, triglycerides; IOP, intraocular pressure.

	Male			Female		
Blepharoptosis	No (n = 3761)	Yes (n = 680)	P-value	No (n = 5003)	Yes (n = 841)	P-value
Age, years	53.3±0.2	59.9±0.6	<0.001	54.4±0.2	65.1±0.5	<0.001
BMI, kg/m^2^	24.1±0.1	24.2±0.1	0.245	23.9±0.1	24.5±0.1	<0.001
Waist circumference, cm	84.9±0.2	86.5±0.4	0.001	80.2±0.2	83.5±0.4	<0.001
Total body fat, %	22.1±0.1	23.2±0.3	0.001	33.6±0.1	34.4±0.3	0.002
Smoking, %			0.389			0.276
Non	17.8(0.7)	18.7(1.8)		92.2(0.5)	91.9(1.2)	
Ex-smoker	39(0.9)	41.2(2.0)		2.8(0.3)	3.9(0.9)	
Current	43.2(1.0)	40.1(2.2)		5.1(0.4)	4.2(0.9)	
Alcohol intake, %			0.011			<0.001
Non-drinker	16.4(0.7)	21.9(1.8)		39.2(0.9)	56.9(2.2)	
Mild~moderate drinker	63.8(1.0)	61.3(2.2)		59.7(0.9)	42.5(2.2)	
Heavy-drinker	19.8(0.8)	16.8(1.8)		1.2(0.2)	0.6(0.3)	
Daily exercise, %	28(1.0)	26.1(2.1)	0.417	24.8(0.9)	21.2(1.9)	0.080
Hypertension, %	43.1(1.0)	49(2.6)	0.023	34.1(0.9)	54.7(2.0)	<0.001
Diabetes, %	13.3(0.7)	20.6(1.7)	<0.001	10(0.5)	18.2(1.8)	<0.001
Metabolic syndrome, %	36.5(1.0)	45.4(2.2)	0.001	36.3(0.9)	59.8(2.0)	<0.001
Family history of eye disease, %	18.5(0.8)	12.5(1.5)	0.002	20.6(0.7)	10.6(1.3)	<0.001
Cataract, %	33(1.3)	54.9(3.0)	<0.001	35.6(1.3)	68.5(2.5)	<0.001
Age related macular degeneration, %	5.2(0.4)	8.7(1.4)	0.003	5.3(0.4)	9.9(1.2)	<0.001
Systolic BP, mmHg	120.9±0.4	122.4±0.9	0.088	119.6±0.4	126.5±0.9	<0.001
Diastolic BP, mmHg	78.7±0.3	77.5±0.5	0.039	75.2±0.2	76.3±0.4	0.014
Fasting glucose, mg/dL	102.8±0.6	105.4±1.0	0.032	97.9±0.4	102.5±1.0	<0.001
Fasting insulin, μIU/ml	9.4±0.1	9.7±0.2	0.377	9.9±0.1	10.5±0.2	0.023
HOMA-IR,	2.4±0.0	2.6±0.1	0.081	2.5±0.0	2.8±0.1	0.001
Total cholesterol, mg/dL	190.9±0.7	188±1.6	0.086	195.5±0.6	198.8±1.4	0.033
HDL-C, mg/dL	48.9±0.2	47.8±0.5	0.053	54.1±0.2	50.4±0.5	<0.001
TG, mg/dL	172.1±3.2	165.7±5.4	0.888	122.8±1.4	150.9±4.9	<0.001
Fat, g	22.1±0.1	23.2±0.3	<0.001	33.6±0.1	34.4±0.3	0.002
Muscle, g	50141±146.3	48631±327.8	<0.001	35837±89.9	35174±196.1	0.001
IOP (Max), mmhg	14.8±0.1	14.7±0.2	0.458	14.4±0.1	14.5±0.1	0.631
Energy/1 day, kcal/d	2299.1±19.0	2040.7±41.5	<0.001	1645±12.7	1444.1±23.9	<0.001
Fat intake/1day, g/d	16.6±0.2	15±0.5	0.002	14.6±0.2	11.8±0.3	<0.001

Because Korea is a single-race nation, all subjects were Asian. The clinical characteristics of the study participants are summarized in Table 1. The cross-sectional analysis included data on 10,285 participants (4,441 men and 5,844 women) aged 40 years or older. The prevalence of blepharoptosis was 14.8% among adult Korean people aged 40 years or older, 15.3% among men and 14.4% among women. As shown in Table 1, although many factors, such as systemic factors (alcohol consumption, hypertension, diabetes, and metabolic syndrome) and ophthalmologic factors (age related macular degeneration, cataract, family history of eye disease, and max intraocular pressure) were thought to be associated with age-related blepharoptosis, after adjustment for aging factors or other confounders, BMI, percentage body fat, and WC were highly associated with age-related blepharoptosis.

### Relationship between obesity and MRD1 (Table 2, Fig 1–3)

**Table 2:** Distribution of obesity parameters according to the severity of blepharoptosis. Data are presented as least square mean ± SE. P for trend is obtained using the general linear model. Model 1 was adjusted for age; model 2 adjusted for family history of eye disease, smoking, exercise, drinking alcohol, total energy intake, and fat intake along with age; model 3 added an adjustment for total cholesterol, and high density lipoprotein cholesterol, diabetes, and hypertension.

MRD1	Male			Female		
	Model 1	Model 2	Model 3	Model 1	Model 2	Model 3
BMI (kg/m^2^)						
≥4mm	24.0±0.1	23.7±0.1	23.9±0.1	23.5±0.1	23.5±0.2	23.8±0.2
3–3.9mm	24.1±0.1	24.0±0.1	24.0±0.1	24.0±0.1	24.0±0.2	24.1±0.2
2–2.9mm	24.2±0.1	24.2±0.2	24.2±0.2	24.2±0.1	24.1±0.2	24.3±0.2
1–1.9mm	24.1±0.2	24.2±0.2	24.1±0.2	24.5±0.2	24.5±0.3	24.5±0.3
<1mm	24.7±0.3	25.3±0.3	25.1±0.3	24.5±0.3	24.6±0.3	24.7±0.3
P for trend	0.041	<0.001	0.005	<0.001	<0.001	<0.001
WC (cm)						
≥4mm	84.5±0.3	84.3±0.4	84.8±0.4	78.9±0.3	81.1±0.6	82.0±0.7
3–3.9mm	84.8±0.3	85.0±0.3	85.0±0.3	80.6±0.3	82.2±0.7	82.6±0.7
2–2.9mm	85.8±0.3	85.9±0.4	85.8±0.5	81.7±0.4	82.3±0.7	82.7±0.8
1–1.9mm	86.0±0.5	86.2±0.5	85.7±0.5	83.5±0.5	83.3±0.7	83.6±0.8
<1mm	88.3±0.8	89.1±0.9	88.0±0.9	83.7±0.7	83.0±1.0	83.5±1.2
P for trend	<0.001	<0.001	0.002	<0.001	<0.001	0.009
Body fat (%)						
≥4.0mm	21.9±0.2	21.5±0.2	21.7±0.2	33.2±0.2	32.9±0.3	33.4±0.4
3–3.9mm	22.0±0.2	21.8±0.2	21.7±0.2	33.8±0.2	33.5±0.4	33.8±0.4
2–2.9mm	22.3±0.2	22.2±0.3	22.0±0.3	33.9±0.2	33.5±0.4	33.8±0.4
1–1.9mm	22.8±0.3	22.6±0.3	22.6±0.3	34.6±0.3	34.0±0.4	34.5±0.4
<1mm	24.4±0.5	24.6±0.5	24.0±0.5	33.9±0.5	33.4±0.5	33.6±0.6
P for trend	0.001	<0.001	0.001	0.001	0.010	0.006

**Fig 1:**
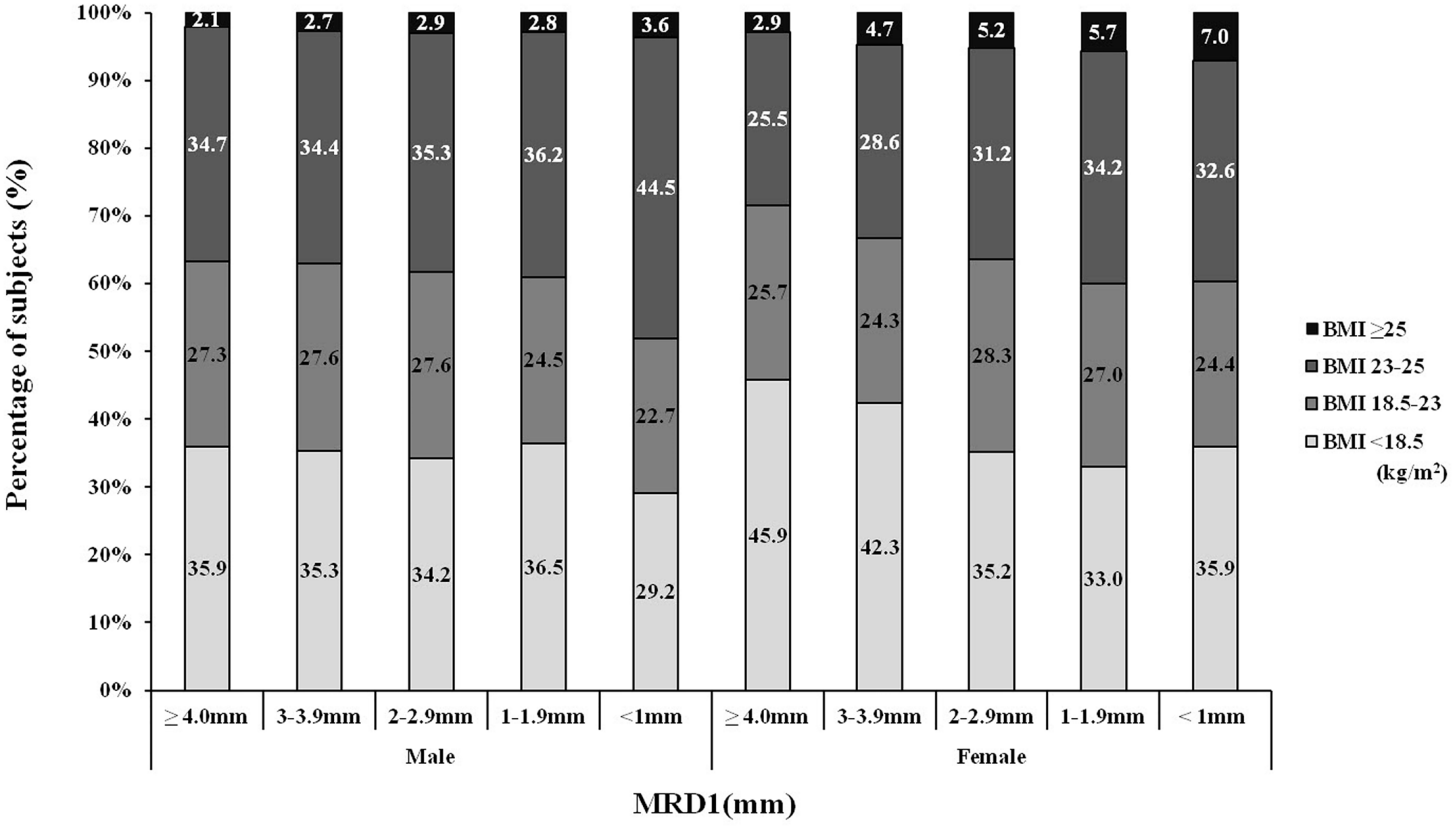
Distribution of the BMI according to MRD1 (male P for trend = 0.121, female P for trend <0.001). As MRD1 decreased (the severity of blepharoptosis worsened), the distribution of subjects with obesity (BMI ≥ 25 kg/m^2^) and those overweight (MRD1 23–25 kg/m^2^) increased.

**Fig 2:**
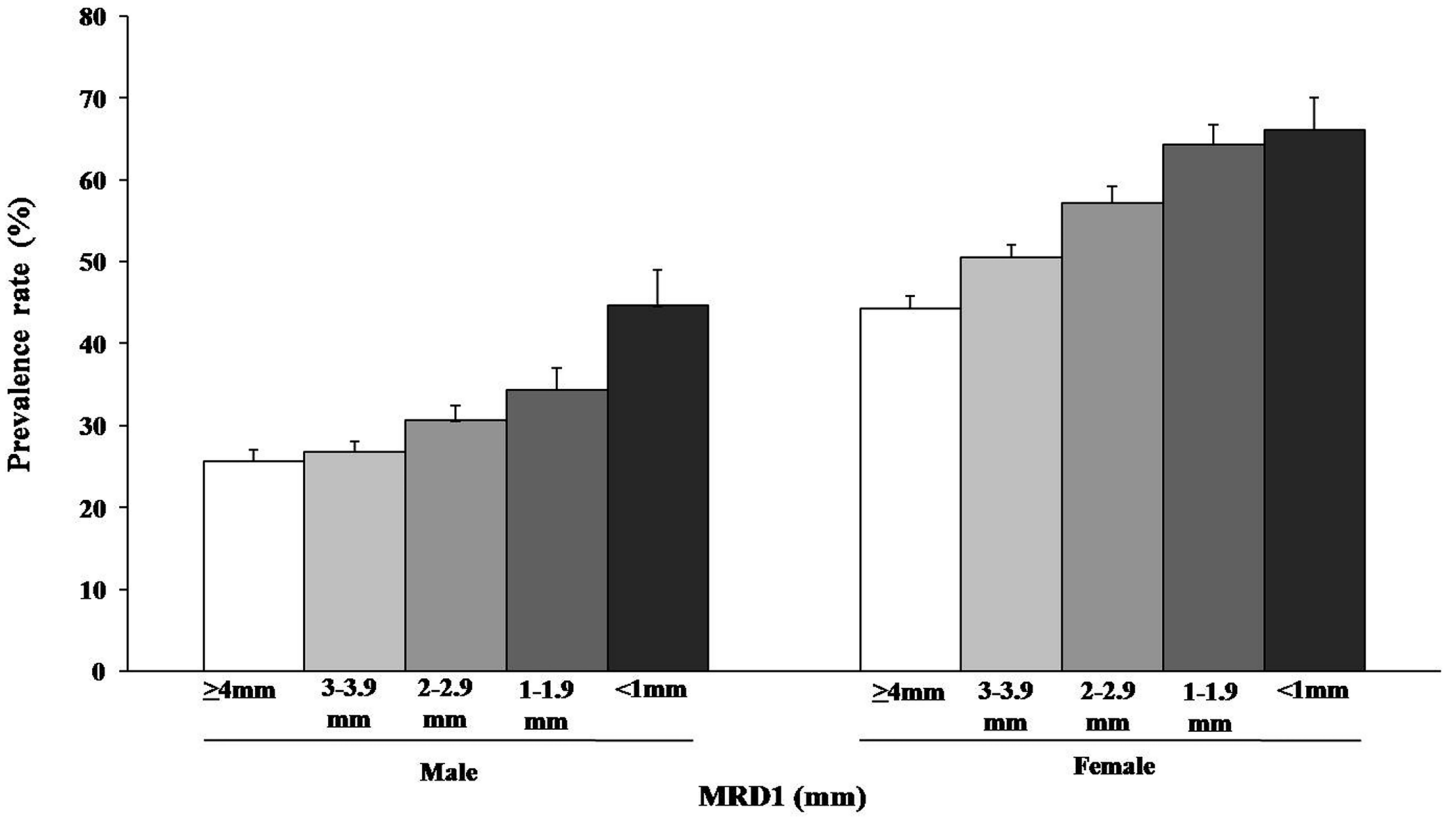
Prevalence of abdominal obesity (male WC ≥ 90 cm, female WC ≥ 80 cm) according to the MRD1. Error bars represent the upper 95% confidence intervals. The prevalence of abdominal obesity increased as MRD1 decreased (the severity of blepharoptosis worsened) (male P for trend < 0.001, female P for trend < 0.001).

**Fig 3:**
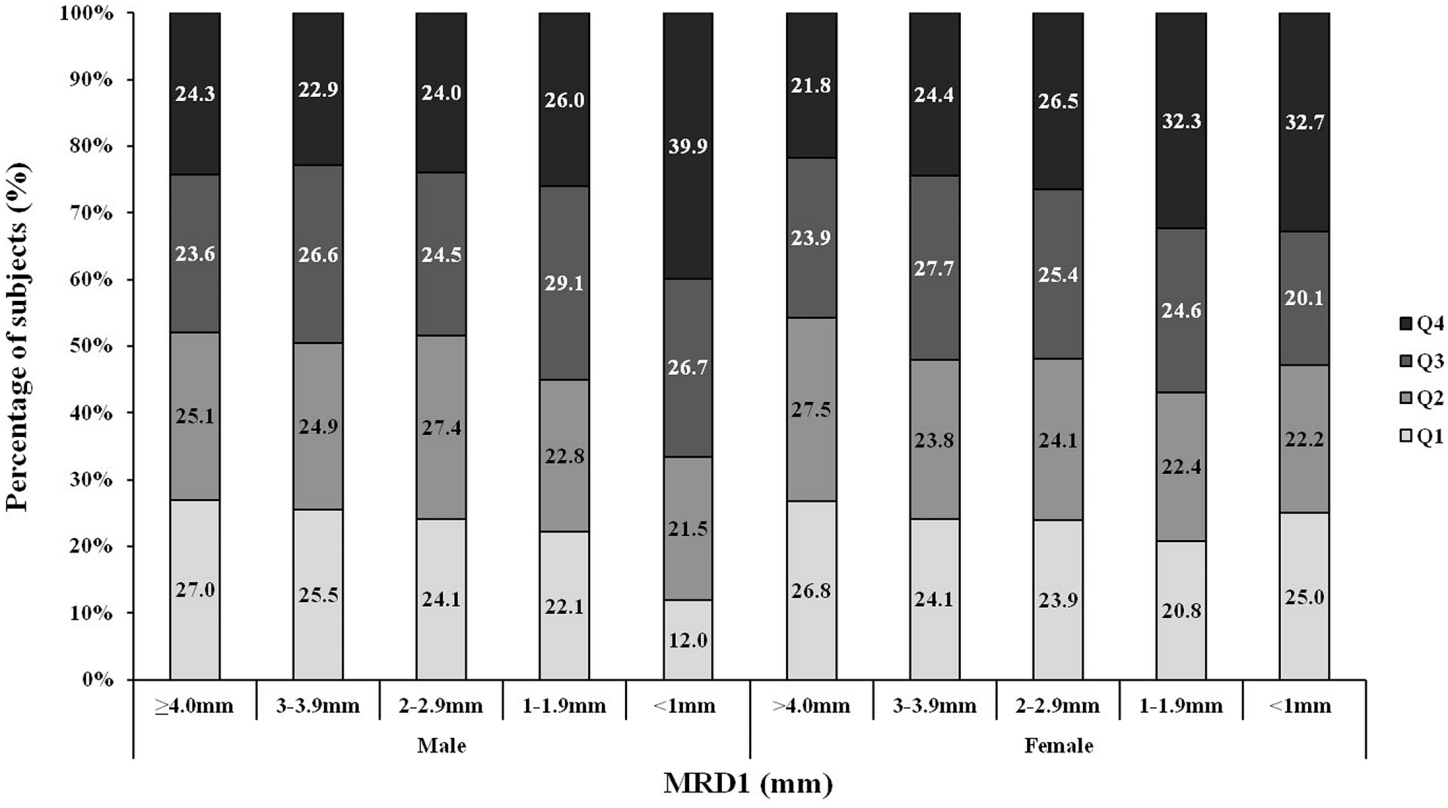
Distribution of subjects into each percentage body fat quartile according to the MRD1. Q1 indicates the lowest 25% percentage body fat, and Q4 indicates the highest 25% percentage body fat. As the MRD1 decreased (the severity of blepharoptosis increased), Q3 and Q4 increased (male P for trend = 0.001, female P for trend <0.001).

We evaluated mean BMI, WC, and percentage body fat according to the severity of blepharoptosis and the comparisons were adjusted for potential confounders including significant parameters in Table 1 which were age (Model 1); family history of eye disease, smoking, exercise, drinking alcohol, total energy intake, and fat intake (Model 2); diabetes mellitus, high blood pressure, total cholesterol and high density lipoprotein cholesterol (Model 3). As seen in Table 2, the mean BMI, WC and percentage body fat increased significantly as the MRD1 decreased (every P for linear trend < 0.05).

We evaluated the distribution of BMI into four categories according to the MRD1, which decreased as the severity of blepharoptosis increased. We found that as the MRD1 decreased, the distribution of subjects with obesity (BMI≥25 kg/m^2^) and overweight (BMI of 23–25 kg/m^2^) increased, and this finding was more prominent in the female population than in the male population (P = 0.121 for males, P < 0.001 for females) (Fig 1). We also found that the prevalence of abdominal obesity (WC: males, ≥ 90 cm; females, ≥ 80 cm) increased as the MRD1 decreased (Fig 2, P < 0.001 for males, P < 0.001 for females). Finally we evaluated the distribution of each body fat quartile according to the MRD1. As seen in Fig 3, as the MRD1 decreased (the severity of blepharoptosis increased), Q3 and Q4 increased (P = 0.001 for males, P < 0.001 for females). Particularly in the male population, the majority of subjects with severe blepharoptosis (MRD1<1) were in Q3 and Q4 (66.6%); the Q3, Q4 proportion was 52.8% among female subjects with severe blepharoptosis.

### Risk of blepharoptosis according to the severity of obesity (Table 3)

**Table 3:** Multiple logistic regression analysis for blepharoptosis according to obesity parameters. P for trend is obtained using linear regression analyses. Model 1 was adjusted for age; model 2 adjusted for family history of eye disease, smoking, exercise, drinking alcohol, total energy intake, and fat intake along with age: model 3 added an adjustment for total cholesterol, and high density lipoprotein cholesterol, diabetes, and hypertension.

	Male			Female		
	Model 1	Model 2	Model 3	Model 1	Model 2	Model 3
BMI, kg/m^2^						
<18.5	0.96(0.74,1.25)	0.91(0.70,1.20)	1.05(0.76,1.44)	0.81(0.64,1.03)	0.82(0.64,1.04)	0.76(0.59,0.97)
18.5–23	1	1	1	1	1	1
23–25	1.32(0.97,1.78)	1.28(0.93,1.77)	1.21(0.87,1.68)	1.13(0.87,1.45)	1.07(0.83,1.39)	0.94(0.71,1.23)
≥25	1.42(0.79,2.51)	1.43(0.81,2.52)	1.48(0.79,2.78)	1.95(1.26,3.03)	1.98(1.26,3.12)	2.14(1.32,3.47)
P for trend	0.011	0.011	0.323	0.001	<0.001	0.002
WC(M≥90, F≥80), cm	1.51(1.19,1.92)	1.58(1.24,2.01)	1.48(1.11,1.97)	1.47(1.18,1.83)	1.40(1.12,1.75)	1.33(1.03,1.71)
Body fat						
Q1	1	1	1	1	1	1
Q2	1.24(0.91,1.71)	1.28(0.94,1.75)	1.19(0.83,1.71)	1.01(0.78,1.31)	0.98(0.75,1.29)	1.06(0.78,1.45)
Q3	1.55(1.14,2.11)	1.77(1.31,2.39)	1.85(1.32,2.60)	1.04(0.79,1.37)	1.04(0.78,1.39)	1.08(0.78,1.50)
Q4	1.86(1.33,2.60)	2.02(1.43,2.87)	2.01(1.34,2.97)	1.47(1.07,2.02)	1.38(0.99,1.90)	1.52(1.06,2.17)
P for trend	0.001	<0.001	<0.001	0.014	0.022	0.018

Table 3 shows that obesity is associated with the risk of blepharoptosis according to the severity of each obesity parameter, which is indicated by the BMI, WC and percentage body fat after adjusting for potential confounding factors. In both genders, there were significantly higher odds for blepharoptosis in subjects with a higher BMI, WC and percentage body fat than in subjects with a lower BMI, WC and percentage body fat, with the exception of the BMI of men in Model 3. Blepharoptosis was significantly associated with general obesity in women [adjusted odds ratio (aOR), 2.14; 95% confidence intervals (CI) 1.32–3.47]; abdominal obesity in men (aOR, 1.48; 95% CI, 1.11–1.97) and women (aOR, 1.33; 95% CI, 1.03–1.71); the highest quartile of percentage BF in men (aOR, 2.01; 95% CI, 1.34–2.97) and women (aOR, 1.52; 95% CI, 1.06–2.17), after adjusting for age (Model 1), family history of eye disease, smoking, exercise, drinking alcohol, total energy intake, and fat intake (Model 2), total cholesterol, high density lipoprotein cholesterol, diabetes, and hypertension (Model 3).

## Discussion

To our knowledge, this is the first large population-based study to examine the association between obesity parameters and age-related blepharoptosis. In this cross-sectional study comprising Korean adults aged 40 years or older, the overall prevalence of age-related blepharoptosis was 14.8% (15.3% for men and 14.4% for women), and there were strong and graded associations between increasing blepharoptosis severity and obesity parameters (BMI, WC, and percentage body fat). In addition, after adjusting for potential confounders, blepharoptosis was significantly associated with general obesity, abdominal obesity, and the highest quartile of percentage BF.

Blepharoptosis, drooping of the upper eyelid, is one of the most common upper eyelid diseases. Symptoms are related to impairment of the superior visual field and central vision in severe cases. Although blepharoptosis is generally known to exist at an MRD1 of less than normal range which is 4~5 mm in Western populations, there are no accurate definitions or available data on the prevalence of blepharoptosis, particularly among Asians or Korean populations [13]. Moreover, depending on race, there are many more differences in facial anatomy among Asians than among Western people [18]. Therefore, we defined blepharoptosis as MRD1< 2 mm as suggested by Shirado and Yoon et al., with consideration of the anatomical differences in Asian eyelids and orbits in their reports [9,13].

In a previous study, Shriado suggested the presence of a relationship between dyslipidemia and age-related blepharoptosis [9], and mentioned that atherogenic dyslipidemia might lead to a circulation disorder that can affect the retinal microvasculature [19], and cause microvascular complications of the eyelid and orbital areas, which are responsible for gradually progressive age-related blepharoptosis [9]. From other studies on the association of blepharoptosis and metabolic disease or endocrine disorders, insulin resistance might be associated, among the unidentified confounders [20–22]. Insulin resistance is related to dyslipidemia as well as to the loss of skeletal muscle mass and strength. Bastiaensen assumed that the cause of acute blepharoptosis in patients with diabetes is mostly oculomotor nerve palsy [23]. In another report, Bastiaensen identified blepharoptosis comorbidity in a large number of patients with diabetes and presumed that this finding may have been related to chronic tissue hypoxia, to which the levator palpebrae muscle is extra-sensitive, and in which thickening of the basal membrane of the capillaries may be an important factor [24].

In many other anatomic and histopathologic studies on age-related blepharoptosis, changes in the eyelid and levator muscle included dehiscence, disinsertion, attenuation and elongation of the aponeurosis [1,2,4], and in some cases, marked fatty infiltration of the levator and Műller muscle [5]. In this report, it was impossible to detect any significant differences in the degree of fatty infiltration of the upper-eyelid elevators or in the status of the aponeurosis between patients with and without obesity. However, we hypothesized that, as total body fat increases and age-related muscular changes including degenerative fatty infiltration occur during the aging process, the fatty changes of the eyelid and levator muscle can change the eyelid position, resulting in the development of blepharoptosis. For all these reasons, we wanted to assess the relationship between age-related blepharoptosis and obesity, as defined by the BMI, WC and percentage body fat.

We also thought some mechanical ptosis can be affected in obese subjects. In the anatomy of Asian eyelid orbital fat protrudes more anteriorly and inferiorly towards eyelid margin due to the more inferior attatchment of the septum to the anterior portion of the levator aponeurosis and tarsus [18]. Therefore, hypertrophied orbital fat in obese subjects may cause mechanical push-down effect on the upper eyelid. These effects are supposed to be pseudoptosis, and should not be considered as true blepharoptosis in company with eyebrow ptosis and dermatochalasis. We could not collect levator function results because of large amounts Data and not verifying data collecting quality. Data collecting team examiners were specially trained and supervised by Committee Survery experts to exclude these pseudoptosis effects.

The present study indicates a possible association between obesity and an increase in the frequency of age-related blepharoptosis. In Table 3, almost all of the parameters of obesity such as BMI, WC and percentage body fat were statistically significant risk factors for age-related blepharoptosis. However, some differences in the obesity parameters were present between the male and female populations. For example, percentage body fat is a more distinct risk factor in males than in females, and BMI is a more distinct risk factor in females than in males. In men in Model 3, individuals with the highest body fat (Quartile 4) are 2.01 times more likely to develop age-related blepharoptosis, whereas, in women in Model 3, individuals with the highest BMI (≥ 25 kg/m^2^) are 2.14 times more likely to develop age-related blepharoptosis. Although the mechanism underlying these differences was unclear, it may be related to sexual (hormonal) differences, because many confounding factors were adjusted for in Model 3. In other Korean population-based studies of metabolic syndrome [25], obesity [26], insulin resistance [11], and the relationship between socioeconomic status and dyslipidemia [27], some sex differences were identified, and in those cases, hormonal differences between the two genders were the probable main factor.

Several limitations of this study should be acknowledged. First, because this was a cross-sectional analysis, causality cannot be inferred from the observed associations. Second, the survey included all patients with blepharoptosis, regardless of their blepharoptosis subtype and all patients’ data were not of merely age-related origin. Finally, information on previous eyelid operations, including ptosis repair surgeries, was unavailable; therefore, there was bias with respect to exclusion of patients with severe blepharoptosis who underwent operations before the data collection period.

Despite these limitations, this is the first study to evaluate obesity parameters as risk factors for age-related blepharoptosis. Furthermore, this is the first nationally representative, population-based study to examine the association of obesity parameters and age-related blepharoptosis among middle-aged and older members of the Korean population. These results suggest possibility of potential study about total body fat, dyslipidemia or metabolic syndrome as a pathogenesis of blepharoptosis.

In conclusion, age-related blepharoptosis is not always a consequence of normal aging. The etiology of this disease may be multifactorial. In addition to the inevitable effects of gravity and senescence, which cause involutional changes of various degrees in eyelid function, obesity should be considered as a possible determinant of age-related blepharoptosis, although further prospective studies are required to confirm a causal. Awareness of this potentially modifiable risk factor, which is also often related to aging, may help in understanding the definitions of the etiology and pathogenesis of age-related blepharoptosis.
